# A Trifluoromethyl Analogue of Celecoxib Exerts Beneficial Effects in Neuroinflammation

**DOI:** 10.1371/journal.pone.0083119

**Published:** 2013-12-11

**Authors:** Alessandra Di Penta, Asako Chiba, Iraide Alloza, Ane Wyssenbach, Takashi Yamamura, Pablo Villoslada, Sachiko Miyake, Koen Vandenbroeck

**Affiliations:** 1 Neurogenomiks Laboratory, University of Basque Country (UPV/ EHU), Zamudio, Spain; 2 Achucarro Basque Center for Neuroscience, Zamudio, Spain; 3 Department of Immunology, National Institute of Neuroscience, National Center of Neurology and Psychiatry, Tokyo, Japan; 4 Department of Immunology, Juntendo University School of Medicine, Tokyo, Japan; 5 IKERBASQUE, Basque Foundation for Science, Bilbao, Spain; 6 Neurotek Laboratory, University of Basque Country (UPV/EHU), Zamudio, Spain; 7 Center of Neuroimmunology, Institute of Biomedical Research August Pi Sunyer (IDIBAPS) – Hospital Clinic of Barcelona, Barcelona, Spain; Catholic University Medical School, Italy

## Abstract

Celecoxib is a selective cyclooxygenase-2 (COX2) inhibitor. We have previously shown that celecoxib inhibits experimental autoimmune encephalomyelitis (EAE) in COX-2-deficient mice, suggestive for a mode of action involving COX2-independent pathways. In the present study, we tested the effect of a trifluoromethyl analogue of celecoxib (TFM-C) with 205-fold lower COX-2 inhibitory activity in two models of neuroinflammation, i.e. cerebellar organotypic cultures challenged with LPS and the EAE mouse model for multiple sclerosis. TFM-C inhibited secretion of IL-1β, IL-12 and IL-17, enhanced that of TNF-α and RANTES, reduced neuronal axonal damage and protected from oxidative stress in the organotypic model. TFM-C blocked TNF-α release in microglial cells through a process involving intracellular retention, but induced TNF-α secretion in primary astrocyte cultures. Finally, we demonstrate that TFM-C and celecoxib ameliorated EAE with equal potency. This coincided with reduced secretion of IL-17 and IFN-γ by MOG-reactive T-cells and of IL-23 and inflammatory cytokines by bone marrow-derived dendritic cells. Our study reveals that non-coxib analogues of celecoxib may have translational value in the treatment of neuro-inflammatory conditions.

## Introduction

Nonsteroidal anti-inflammatory drugs are indicated for the treatment of a variety of chronic inflammatory diseases and act by inhibiting prostaglandin H synthase (also known as cyclooxygenase, COX). Two forms of this enzyme are known: COX1 that is expressed constitutively in most tissues and plays a role in the protection of the gastrointestinal mucosa, renal hemodynamics, and platelet thrombogenesis, and COX2 that is inducible and expressed in cells involved in inflammation [1]. Selective COX2 inhibitors (i.e., celecoxib, rofecoxib, and valdecoxib) have been developed for the treatment of inflammatory diseases [2] minimizing gastrointestinal adverse reactions caused by COX-1 inhibition. 

Celecoxib has been demonstrated to act via both COX2-dependent and -independent pathways, both associated with potent anti-tumour effects [3,4]. Recently, we have shown that both celecoxib and its trifluoromethyl analogue, TFM-C (4-[5-(4-trifluoromethylphenyl)-3-(trifluoromethyl)- 1*H*-pyrazol-1-yl]benzenesulfonamide) that displays 205-fold lower COX2-inhibitory activity, can inhibit secretion of (hetero)dimeric cytokines belonging to the interleukin-12 (IL-12) family (including IL-23 and p40 homodimers) with similar IC_50_’s. Using recombinant cell lines, TFM-C caused Ca^2+^-dependent chaperone-mediated cytokine subunit retention in the endoplasmic reticulum (ER) coupled to degradation via the ER stress protein HERP [5,6]. This observation provided a translational extension of recent findings in our lab of functional chaperone interaction peptide motifs conserved in cytokines as well as of multiple ATP-dependent cytokine-chaperone interactions during ER transit of cytokines [7-9]. Perturbance of ER Ca^2+^ by celecoxib and non-coxib analogues triggers a cellular response known as “unfolded protein response” (UPR) [4,10,11]. The UPR has a dual role in the cell survival: in normal conditions works as a pro-survival response mediated by the blockage of protein translation and activation of transcription factors that can restore the ER function to its normal physiological state. However, if ER stress persists, its pro-survival function can switch to a pro-apoptotic program, finally leading to cell death [12,13]. 

COX-2 inhibitors such as indomethacin or rofecoxib exert beneficial effects in EAE; the latter, for example, by modulating Th1/Th2 responses [14,15]. Recently, we have shown that celecoxib inhibits EAE [16]. In particular, we found that celecoxib in contrast to other COX-2 inhibitors such as nimesulid, prevented EAE by inhibiting the infiltration of inflammatory cells into the central nervous system, MOG-specific Th1 cytokine production and expression of adhesion molecules and MCP-1 [16]. This protective effect was not mediated via COX2 inhibition since it was also observed in COX2-deficient mice [16]. We have demonstrated that celecoxib and its analogue TFM-C exerted beneficial effects in two models of arthritis: collagen-induced arthritis (CIA) and collagen antibody-induced arthritis (CAIA). In particular, TFM-C, more so than celecoxib, inhibited the severity of CIA and CAIA by suppressing the activation of mast cells and the production of inflammatory cytokines by macrophages [17]. Nevertheless, celecoxib and other COX2 inhibitors display adverse effects that may compromise further implementation in clinical practice. Increased risk for myocardial infarction and stroke in drug recipients have been reported [18-22]. Comparison of cardiovascular risks profiles of celecoxib vs the non-selective nonsteroidal anti-inflammatory drug naproxen and ibuprofen is currently addressed in the PRECISION trial (http://clinicaltrials.gov/show/NCT00346216). Suppression of prostacyclin (PGI2) and prostaglandin E2, two COX-2-derived products, may provide protective constraint mechanisms on processes such as thrombogenesis and atherogenesis [23]. In rodents, celecoxib predisposes to platelet activation and arterial thrombosis by suppressing PGI2 [24]. Similarly, selective inhibition of COX-2 can enhance platelet–vessel wall interactions and platelet adhesion *in vivo* increasing the risk of thrombosis [25]. 

We hypothesized that TFM-C may constitute a new drug candidate that retains the beneficial effects of celecoxib in the EAE model while its much decreased COX2 inhibitory activity would render it less adverse in terms of cardiovascular risk. In this study, we have analyzed the effect of TFM-C on cytokine secretion, demyelination, and axonal damage in mice cerebellar organotypic cultures, a model of neuroinflammation, and assessed its activity in the EAE model.

## Materials and Methods

### Ethics statement

Animals were handled in accordance with the European Communities Council Directive (Directive 2010/63/EU), the Spanish regulations for the procurement and care of experimental animals (RD 53/2013, February 1st), and the study was approved by the Ethical Committee on Animal Research of the University of Basque Country (UPV/EHU). EAE experiments were approved by the Institutional Animal Care and Use Committee of the National Institute of Neuroscience (Tokyo, Japan). 

### Materials and animals

All animal experiments in this study were performed using C57BL/6J mice (Harlan Laboratories, Italy). C57BL/6J Jcl mice used in EAE experiments were purchased from CLEA Japan, Inc. Mice were maintained in a temperature-controlled environment with food and water *ad libitum* at 12-hour light/12-hour dark cycle. The animals used in this study were 8 weeks old for EAE experiments and 8 days old for cerebellar organotypic cultures experiments. TFM-C was synthesized by Onyx Scientific (Sunderland, UK). All antibodies used in this work are indicated in Table 1. 

**Table 1:** List of primary antibodies used for immunofluorescence (IF) and western blot (WB) studies.

**Antigen**	**Description**	**Dilution**	**Company**
**ARMET**	ARMET antibody, Rabbit	1:2000 (WB)	AbCam
**HERP**	Homocysteine-induced endoplasmic reticulum protein, Mouse	1:200/1:400 (IF) 1:2000 (WB)	Hirabayashi Y [6].
**Iba1**	Ionized calcium binding adaptor molecule 1: anti-Iba 1, Rabbit	1:400/1:500 (IF)	Wako
**iNOS**	Inducible nitric oxide synthase: purified rabbit anti-iNOS/NOS type II	1:200 (IF) 1:500 (WB)	BD Bioscience
**LC3B**	LC3B (D11) XP^TM^ Rabbit mAb	1:1000 (WB)	Cell Signaling
**MBP**	Myelin Basic Protein; Rat anti-MBP (82-87) antibody	1:200 (IF)	Serotec
**NFH**	Neurofilament heavy (phosphorylated and non-phosphorylated NfH): rabbit polyclonal antiserum against the 200 kD Neurofilament Heavy	1:200 (IF)	AbCam
**NFL**	Neurofilament light C28E10, Rabbit mAb	1:200 (IF)	Cell Signaling
**PARK2**	Rabbit polyclonal to Parkin	1:750 (WB)	AbCam
**RAB24**	Purified Mouse Anti-Rab24	1:1000 (WB)	BD Bioscience
**SMI32**	non-phosphorylated neurofilament heavy SMI32, Mouse	1:200 (IF)	Stenberg
**TNF-α**	Anti-Murine TNF-alpha, Rabbit	1:300 (IF)	MBL
**Tubulin**	α-Tubulin Antibody [HRP], mAb, Mouse	1:2000	GenScript

### Induction and clinical assessment of EAE

C57BL/6J Jcl (B6) female mice (n = 5-6 per group, 7-8 weeks old) were immunized subcutaneously at the base of the tail with 100 µg of myelin oligodendrocyte glycoprotein 35-55 (MOG35-55) peptide (amino acid sequence, MEVGWYRSPFSRVVHYRNGK, derived from mouse MOG) dissolved in 0.1 ml phosphate buffered saline (PBS) and 0.1 ml CFA containing 1 mg of Mycobacterium tuberculosis strain H37Ra (*Mtb* H37Ra). Shortly after immunization and 48 h later, the mice were injected i.p. with 200 ng of Bordetella pertussis toxin (List Biological Laboratories). Mice were randomly assigned to three treatment groups receiving intraperitoneal injections of TFM-C or celecoxib at doses of 10 µg/g, or control vehicle every other day from the day of immunization. In the control group, the sample size is n=5-6 per group (16 animals in total) and in the TFM-C-treated group n=4-5 animals per group (14 animals in total). The experiment was repeated three times in order to respect the directives for reduction of the number of animals in animal experiments. Only 5 animals were used in the celecoxib-treated group because we have assessed the effect of celecoxib in a previous study [16]. Clinical scores of EAE were assigned daily as follows: 0 = normal; 1 = weakness of the tail and/or paralysis of the distal half of the tail; 2 = loss of tail tonicity; 3 = partial hind limb paralysis; 4 = complete hind limb paralysis; 5= forelimb paralysis or moribund; 6 = death. The EAE scoring was conducted by personnel unaware of treatment-group assignments. Mice were sacrificed with lethal dose of diethyl ether.

### Cerebellar organotypic cultures

The cerebellar slice culture was based on published protocols [26,27]. Organotypic slice cultures were prepared from 8-day old C57BL/6 mice. Cerebella were cut at 350 µm with a McIlwain Tissue Chopper (The Mickle Laboratory Engineering Co. LTD.) and three slices were plated on Millicell-CM culture inserts. From 10 mice we obtained approximately 90 slices. Cultures were incubated at 37°C and 5% CO_2_ in 50 % basal medium with Earle’s salt, 25% Hank’s buffered salt solution, 25% inactivated horse serum, 5mg/ml glucose, 0,25mM L-glutamine and 25µg/ml Pen/Strep (Invitrogen). Cerebellar slices were maintained in culture for 7 days and then pre-treated with 50 µM of TFM-C for 2 hours followed by stimulation with 15 µg/ml of lipopolysaccharide (LPS; Sigma L4391) for 6 and 24 hours, or as indicated. 

### BV2 cell line

BV2 cells were provided by Prof. Carlos Matute (University of the Basque Country, Leioa, Spain) [28] and were maintained in Dulbecco's Modified Eagle's medium (DMEM) containing 5% heat inactivated Fetal Bovine Serum (FBS), 4 mM L-Glutamine (SAFC biosciences), 20 mM Hepes (Sigma) and 25µg/ml Pen/Strep (Invitrogen) antibiotics at 37°C in a humidified chamber with 5% C0_2_. Before treatment, cells were washed twice with DMEM, then pre-incubated with TFM-C (30 µM or 50 µM) for 2 hours and stimulated with 1 µg/ml LPS (Sigma L4391) for 3, 6, 12 and 24 hours. 

### HEK-293 cell line and PCR arrays

Human embryonic kidney-293 cells (HEK-293; Invitrogen), were cultured in DMEM (Sigma) supplemented with 10 % fetal bovine serum (Sigma), 2 mM L-glutamine (Invitrogen) and 25µg/ml Pen/Strep (Invitrogen). Cells were washed twice with DMEM and incubated with TFM-C (50 µM) for 3, 6, 12 and 24 hours. RNA was extracted and reverse transcribed using RT^2^ First Strand Kits (SABiosciences, address), followed by profiling of the expression of 84 key genes each belonging to UPR, Ubiquitination and Autophagy Pathway RT^2^ Profiler PCR Arrays (SABiosciences, address). Data was analyzed using the manufacturer’s PCR Array Data Analysis Software.

### Total protein extraction and Western Blot

Three cerebellar slices, HEK-293 and BV2 cells were homogenised by stroke dounce homogenization in 60 μl of ice-cold RIPA buffer (150mM NaCl; 50mM Tris-Cl, pH 7.5; 1% NP-40; 0.5% DOC; 0.1% SDS) and the lysate was centrifuged at 15.000 x g for 10 min at 4°C. Total protein concentration was estimated by the Bradford assay (Bio-Rad). 10 µg of total protein from cerebellar slices, BV2 or HEK-293 cells were separated by SDS-PAGE on Bio-Rad TGX Stain-Free precast gels. The gels were exposed to UV irradiation for 5 min and then total protein was transferred onto a nitrocellulose membrane (Bio-Rab). UV irradiation activates a covalent reaction between the trihalocompound and tryptophan residues on the proteins in the gel, resulting in UV induced fluorescence (Figure S5). Total protein was visualized with ChemiDoc (Bio-Rad) and quantified using Image Lab 4.0 software. The filters were pre-wetted in blocking buffer (5% dried non-fat milk or 2% casein, 20 mM Tris pH 8, 300 mM NaCl, 0.1% Tween-20) and then hybridized for 2h or O.N. with primary antibodies in blocking buffer (Table 1); the secondary antibodies (HRP-conjugated anti-mouse or anti-rabbit from Cell Signaling) were used at a dilution of 1:2000. 

### RNA isolation and Quantitative RT-PCR (QPCR)

Cerebellar organotypic cultures, HEK-293 and BV2 cells were lysed with TRI reagent (Sigma). Chloroform was added for phase separation with RNA remaining in the aqueous phase. RNA was precipitated with 2-propanol and the pellet resuspended in DNA/RNA free water (Invitrogen). RNA was quantified using Nanodrop 2000c spectrophotometer (Thermo Scientific). 200 ng of mice organotypic cultures, HEK-293 and BV2 cells RNA were reverse-transcribed to cDNA using random primers according to manufacturer’s protocol (Applied biosystems). QPCR was performed with the Supermix for SsoFast EvaGreen (Biorad) on a 7500 Fast Real-Time PCR System (Applied Biosystems). For each target gene, QPCR QuantiTect Primer Assay were used (Qiagen). Expression levels for the transcripts of interest were normalized to that of endogenous glyceraldehyde 3-phosphate dehydrogenase or hypoxanthine phosphoribosyltransferase 1 (Hprt1). The data were calculated as percentage fold expression or 2^-∆∆Ct^ relative to the average of the untreated control group, unless indicated otherwise.

### Quansys Q-Plex^TM^ Array Chemiluminescent and ELISA

For Q-Plex system analysis, 30 µl of medium from mice cerebellar organotypic cultures treated with LPS or LPS/TFM-C were used. Mouse Cytokine Stripwells (16-plex) were used following the manufacturer’s instructions (Quansys Bioscience). The chemiluminescent signal was acquired with Bio-Rad ChemiDoc camera and the image was analyzed with Q-View Software (Quansys Bioscience). BV2 cells were pre-treated with TFM-C (30 µM or 50 µM) for 2 hours and then stimulated with LPS for different periods of time (0, 3, 6, 12 and 24 hours) and the culture supernatants were collected to quantify the secreted IL-1β, tumor necrosis factor (TNF-α) and IL-6. Mouse ELISA Kits were used according to the manufacturer’s instructions (eBioscience, BD Bioscience, R&D Systems). 

### Immunofluorescence microscopy

Cerebellar slices were fixed with 4% paraformaldehyde (PFA) for 40 min, washed with PBS for 10 min, and blocked at RT for 2 hours in 10% normal goat serum (NGS; Vector Laboratories) and 0.5% Triton X-100 in PBS. The slices were incubated overnight at 4°C with the distinct primary antibodies (Table 1) in blocking solution (10% NGS and 0.3% Triton X-100 in PBS). The slices were washed and incubated in blocking solution containing the secondary antibody mixture and washed three times with 0.1% Triton X-100 in PBS. BV2 cells were fixed with 4% PFA for 20 min, washed with PBS for 10 min, permeabilized for 20 min with 0.2% Triton X-100 in PBS and blocked at RT for 30 min in 10% FBS ( Invitrogen) in PBS. The cells were incubated 1 hour with the Iba1 and HERP (1:400; kindly supplied by Dr. Yasuhiko Hirabasyashi, Tohuku University, Japan) or TNF-α primary antibodies in blocking solution (10% FBS in PBS). After washing, the cells were incubated in blocking solution containing the secondary antibody mixture, washed three times with PBS and incubated with DAPI (1:50000; Molecular Probes) in PBS. The secondary antibodies used were mouse IgG Cy2-linked, rabbit IgG Cy3-linked (from goat, 1:200; GE Healthcare) and goat anti-rat IgG Alexa Fluor 488 (1:200; Molecular Probes). The slices and coverslips were mounted in Gel/Mount anti-fading mounting medium (Biomeda) and pictures were captured by confocal scanning microscopy (Olympus Fluoview FV500) from single images all through the whole tissue or cells (but avoiding the surface of the culture in contact with air). 

### DAPI and Propidium iodite (PI) staining

12 hours before LPS/TFM-C (3, 6, 12 and 24 hours) treatment BV2 cells were incubated over night with PI (250 ng/ml; Fluka) and then fixed with 4% PFA. The cells were washed three times with PBS and then incubated with DAPI (1:50000; Molecular Probes) in PBS. Coverslips were embedded in Fuoro-Gel (Electron Microscopy Science). Images were recorded using the confocal microscope and analyzed using the ImageJ program (version 1.40). 

### Flow cytometry of BV2 cells

The expression of intracellular TNF-α was evaluated by cytofluorometry. 1x10^6^ cells were plated and after 24 hours treated with LPS (1µg/ml) or pre-treated with TFM-C for 2 hour and then stimulated with LPS/TFM-C for 3, 6, and 24 hours. The cells were detached with trypsin, washed in PBS, pelleted at 300 g for 10 min and fixed with PFA 2% for 20 min. Then, cells were permeabilized with 0,2% Triton in PBS for 20 min, blocked with 10% NGS in PBS for half hour, and incubated with primary antibody TNF-α (1:100; MBL) for 1 hour in blocking solution. After washing, cells were incubated with secondary antibody (1:500 goat anti-mouse AlexaFluo 488; LifeTechnology) in blocking solution for 30 min in darkness. Following further washes, cells were pelleted and the fluorescence was analyzed using a Gallios cytometer (Beckman Coulter) at excitation/emission wavelengths of 488/515-535 nm.

### Astrocyte-enriched cell culture

Primary cultures of cerebral cortical astrocytes were prepared as described previously by McCarthy and de Vellis (1980) [29]. Briefly, forebrains were removed from the skulls, the meninges were carefully excised under a dissecting microscope, and the cortices were isolated. Tissue was incubated for 15 min in Hank’s Balanced Salt Solution (HBSS) with tripsine and DNAase, the reaction was stopped with DMEM supplemented with 10% FBS and centrifuged to remove cellular debris. Cells were resuspended in Iscove’s Modified Dulbecco’s Medium supplemented with 10% FBS and antibiotic/antimycotic solution, and the cells were dissociated by passage through needles of decreasing diameter (21 G and 23 G) 10 times. Cells were re-centrifuged and then resuspended in Iscove’s Modified Dulbecco’s Medium supplemented with 10% FBS and antibiotic/antimycotic solution. Cells were seeded into poly-D-lysine-coated flasks, and maintained in culture at 37°C and 5% CO_2_. The culture medium was renewed the day after seeding, and twice a week thereafter. After 8 days, cultures were shaken (3 hours, 350 rpm) to deplete microglial cells and were trypsinized and plated onto poly-D-lysine-coated, 24-well plates.

### Statistical analysis

All experiments were performed at least three times, and control cultures were time-matched with testing cultures. The values were expressed as the means ± SEM. ANOVA or Student’s t-tests were used to determine statistical significance, as indicated, and all analyses were performed using SPSS 15.0 software (IBM). EAE clinical or pathological scores for the group of mice are presented as the mean group clinical score +SEM, and statistical differences were analyzed with a non-parametric Mann-Whitney U-test.

## Results

### TFM-C modulates cytokine release and suppresses oxidative stress and axonal damage in mouse celerebellar organotypic cultures

**Figure 1:**
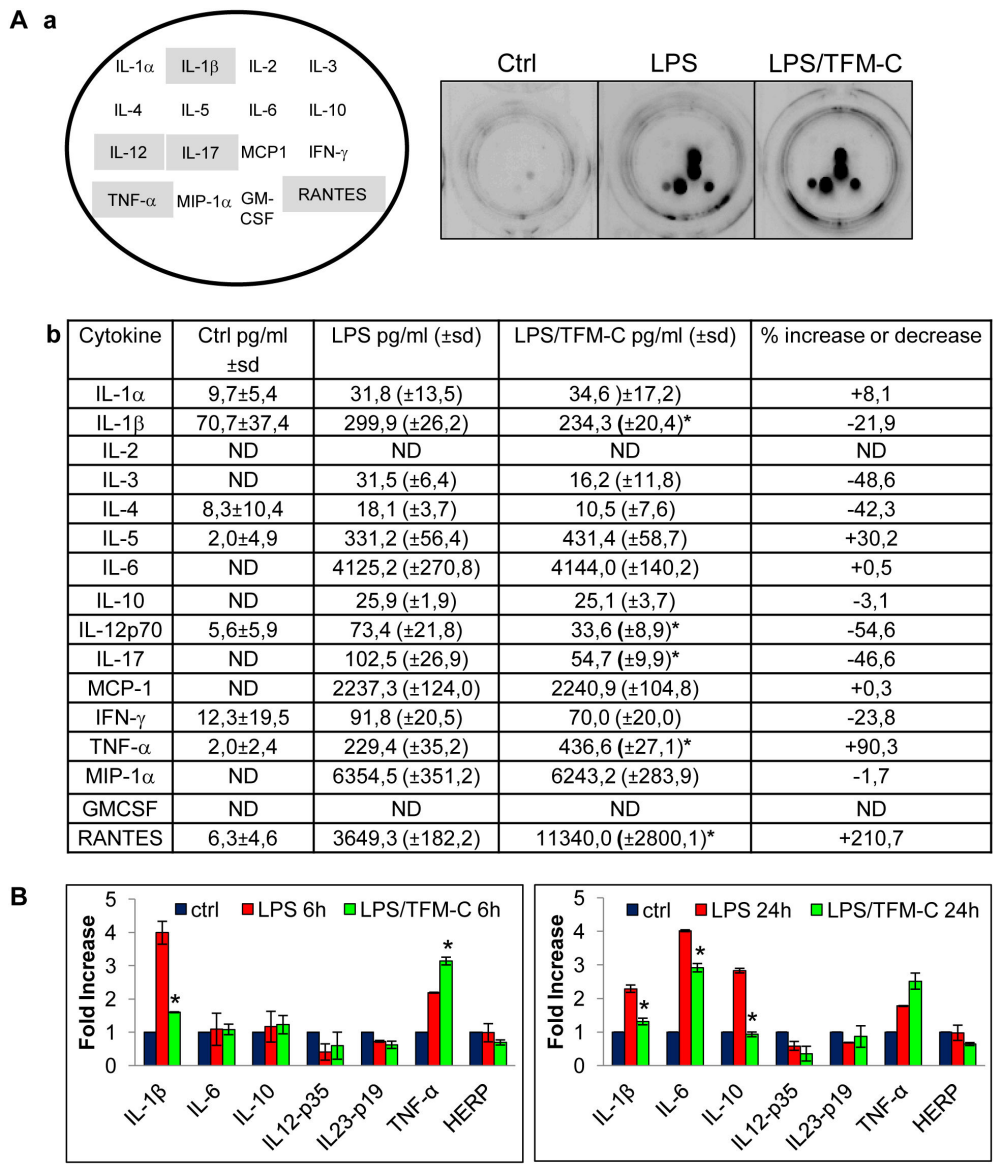
Effect of TFM-C on cytokine production in organotypic cerebellar cultures. A) After 7 *DIV* organotypic cultures were treated with TFM-C (50µM) for 6h and then stimulated with 15µg/ml LPS for 24h in presence of TFM-C. Panel a) Lay-out of cytokine-specific antibody spots in the 16-plex cytokine Stripwell array (left image) and visualization of cytokine-specific chemiluminescence in culture medium of LPS-treated organotypic cultures in the absence or presence of TFM-C (right images). Cyotkine levels significantly affected by TFM-C are highlighted in grey. Panel b) pg/ml of cytokines were indicated. sd: standard deviation. ND: not determinable. B) Effect of TFM-C on IL-1β, IL-6, IL-10, IL-12p35, IL-23p19, TNF-α and HERP mRNA in organotypic cultures stimulated by LPS for 6h and 24h in presence or absence of 50µM TFM-C. The levels of mRNA are shown as *n*-fold increase compared with baseline level (-) and normalized to those of the housekeeping gene *Hprt1*. Asterisks indicate significant differences at *<*P* 0.05 compared with LPS control by ANOVA test.

The effect of TFM-C on cytokine release was assessed in a model of neuroinflammation consisting of mice cerebellar organotypic cultures stimulated with LPS, by means of a multiplex ELISA-based Q-Plex assay system (see Materials and Methods). Of 16 assessed cytokines, 5 were identified of which LPS-induced secretion was modulated by TFM-C (Figure 1A). While LPS challenge induced varying levels of the pro-inflammatory cytokines IL-1α, IL-1β, IL-3, IL-5, IL-6, IL-10, IL-12, IL-17, MCP-1, IFN-γ, TNF-α, MIP-1α and RANTES (Figure 1A), TFM-C treatment significantly decreased IL-1β, IL-12 and IL-17 release and increased TNF-α and RANTES. mRNA levels of a subset of these cytokines were also measured in mice organotypic cultures treated with LPS or LPS/TFM-C. HERP was included as a marker for ER stress that is inducible by TFM-C [6,9]. TFM-C treatment decreased IL-1β mRNA expression levels at 6 and 24 hours, but those of IL-6 and IL-10 only at 24 hours (Figure 1B). In contrast, TNF-α mRNA levels were significantly higher after 6 hours of TFM-C treatment and this trend persisted at the 24 h time-point (non-significant). This mirrored the increased levels of secreted TNF-α demonstrated in Figure 1A. No significant changes were observed for IL-12p35, IL-23p19 and HERP mRNAs. 

**Figure 2:**
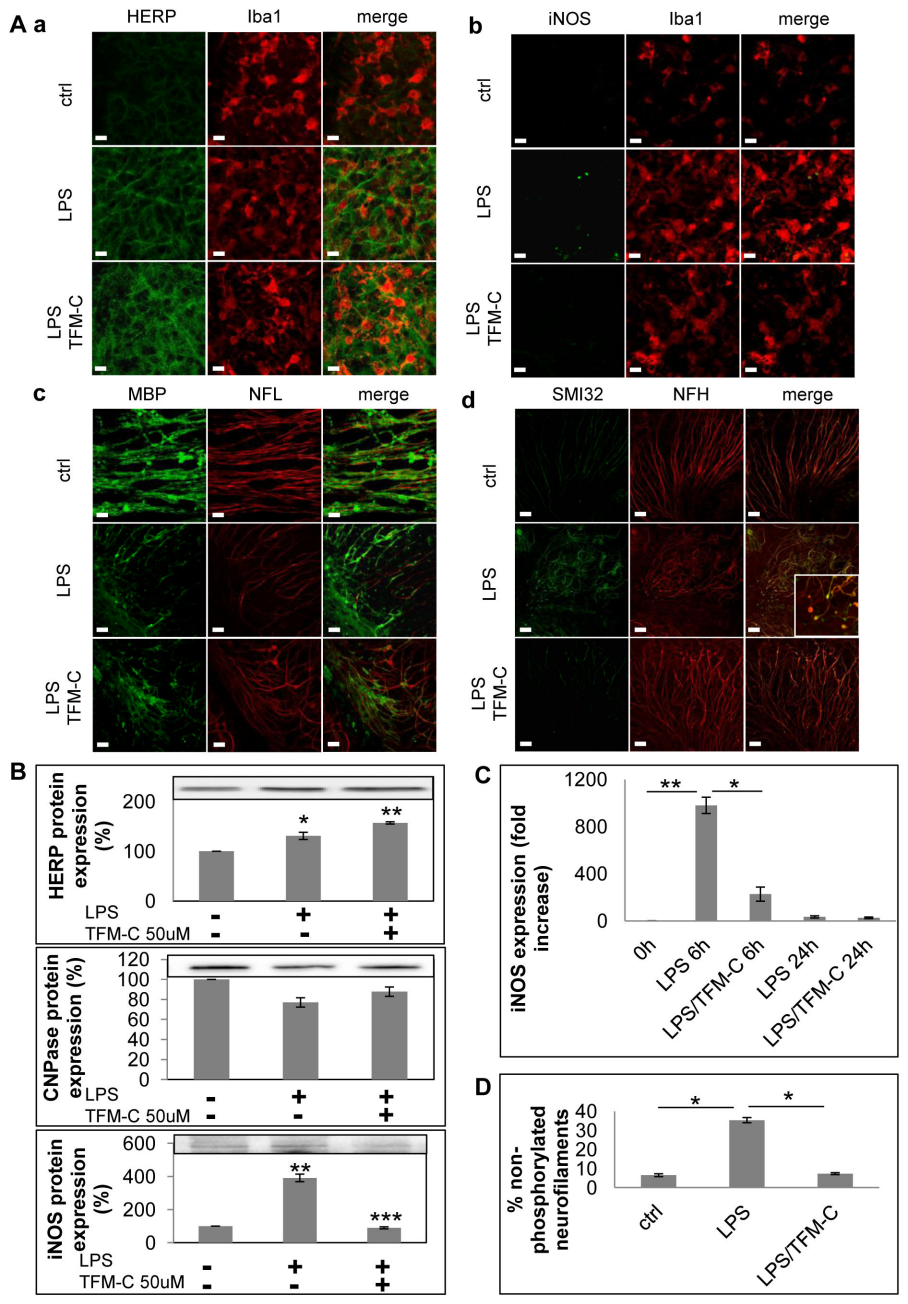
Effect of TFM-C on ER stress, oxidative stress, demyelination and axonal damage in organotypic cerebellar cultures. A) Organotypic cultures were stimulated with LPS for 24h or pre-treated with TFM-C (50µM) for 2h and then stimulated with LPS/TFM-C for 24h. Panel a-b: immunostaining for Iba1 (red) and HERP or iNOS (green). Panel c: immunostaining for NFL (red) and MBP (green); Panel d: immunistaining for total neurofilament-heavy (NFH; red) and non-phosphorylated neurofilament (SMI32; green). In the white box inset are shown axonal spheroids and occurrence of axonal transection (end-bulbs). Scale bar 10µm. B) 10µg of total protein were loaded for HERP, CNPase and iNOS Western blot analysis. Quantification of band intensity was calculated in the graphs below after normalization for total protein loaded. Results are expressed as percentage compared to the LPS only (100%). Error bars indicate the standard error. *P<0.05, **P<0.001, ***P<0.001 by ANOVA test. C) After 7 *DIV* organotypic cultures were treated with TFM-C for 2h and then stimulated with 15µg/ml LPS for 6 and 24h. iNOS mRNA expression was analyzed by quantitative PCR. The levels of mRNA are shown as *n*-fold increase compared with the level of baseline condition (-) and normalized to those of the housekeeping gene *Hprt1*. All values represent the averages of three independent experiments. Error bars indicate the standard error. *P<0.05, **P<0.01, ***P<0.001 by ANOVA test. D) Percentage of non-phosphorylated neurofilaments with respect to total neurofilaments in cerebellar cultures stimulated for 24h with LPS in presence or absence of TFM-C. Error bars indicate the standard error. *P <0.05 by ANOVA test.

Next we scored the effect of TFM-C on LPS-induced oxidative stress, demyelination and axonal damage in the organotypic model [30-33] using immunofluorescence, immunoblot and QPCR. The analyzed markers included iNOS for oxidative stress, CNPase for demyelination, SMI32 for axonal damage, and HERP for ER stress. Immunofluorescence showed that TFM-C increased HERP protein expression in microglia identified with Iba1, and upregulated HERP protein in the total culture (Figure 2A, panel a; Figure 2B). Axonal damage was visualized by dual immunostaining for both total (phosphorylated and non-phosphorylated) NFH and non-phosphorylated NFH (SMI32; Figure 2A, panel d). In response to LPS, non-phosphorylated NFH was found to accumulate at 3.5 higher levels compared to total NFH, suggestive for induction of axonal dysfunction. Furthermore, LPS-induced axonal damage was visible via formation of swollen structures (beading and spheroids; white box in Figure 2A, panel d) associated with impaired axonal transport and transection [34]. TFM-C prevented formation of swollen structures and maintained LPS-induced non-phosphorylated neurofilament levels to those of baseline control (Figure 2D). In contrast, TFM-C treatment had no effect on demyelination assessed by MBP/NFL immunofluorescence (Figure 2A, panel c) or CNPase expression (Figure 2B). Finally, pretreatment with TFM-C prior to LPS challenge strongly reduced iNOS expression determined by immunofluorescence, Western Blot and QPCR (Figure 2A, panel b; Figure 2B & C). TFM-C did not affect expression of MHCII (Figure S1). Taken together, these results indicate that TFM-C partially counteracted LPS-induced effects reminiscent of an anti-inflammatory, anti-oxidant and neuroprotective mode of action. 

### TFM-C suppresses cytokine release and induces UPR / ER stress in BV2 microglia cells

**Figure 3:**
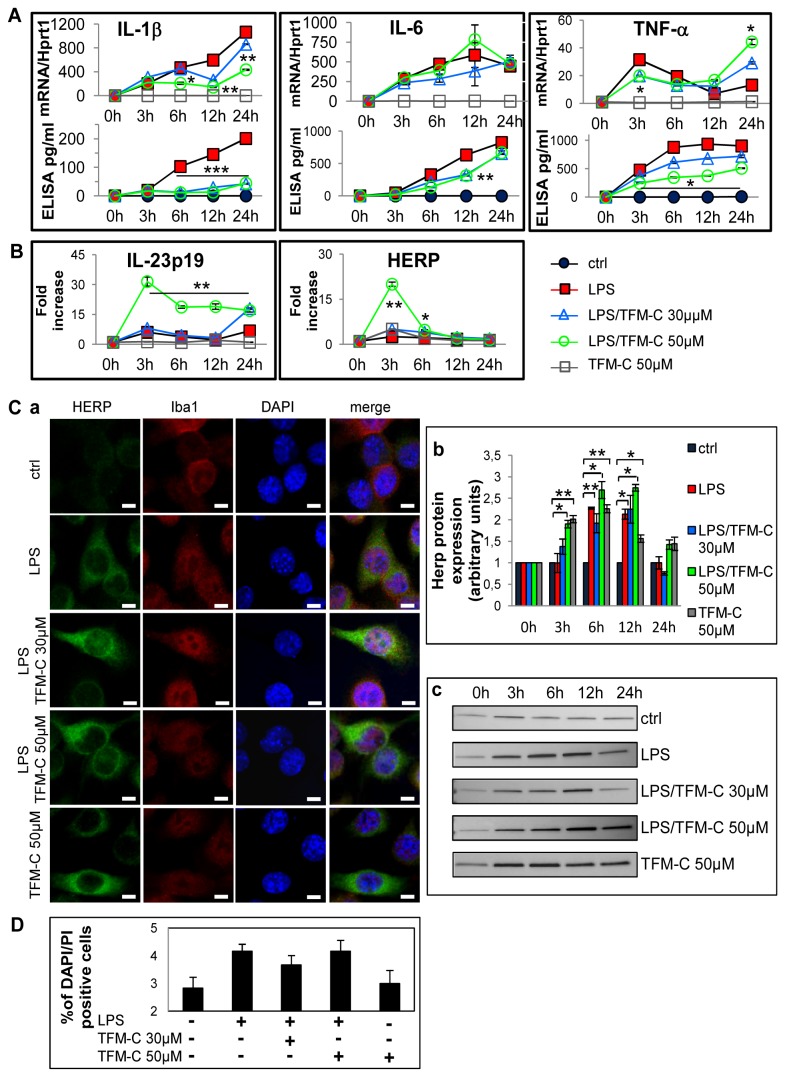
Effect of TFM-C on HERP and cytokine mRNA and protein production in BV-2 cells. BV2 cells were treated with TFM-C (30 or 50µM) for 2h and then stimulated with LPS (1µg/ml) for different times in presence or absence of TFM-C. A) Quantification of the kinetics of mRNA production and cytokine secretion (IL-1β, IL-6 and TNF-α). All values represent the averages of three independent experiments. Lower graphs represent cytokine-specific mRNA quantified by QPCR, while the upper graphs represents amount of secreted cytokine quantified using specific ELISA kits. Asterisks indicate significant differences at *P< 0.05, **P<0.01, ***P<0.001 between TFM-C-treated and LPS-treated cells at each time point using ANOVA test. B) Effect of TFM-C (30 and 50µM) on IL-23p19 and HERP mRNAs in BV2 cells stimulated with LPS. The levels of mRNA levels are shown as fold increase. Asterisks indicate significant differences at *P < 0.05 and **P<0.01 compared with LPS only using ANOVA test. C) Effect of TFM-C on HERP protein expression. BV2 cells were treated with TFM-C (50µM) or pretreated with TFM-C for 2h (30 and 50µM) and then stimulated with LPS in presence of TFM-C. Panel a) Immunofluorescence for HERP (green), Iba1 (red) and DAPI (blue) at 12h of LPS/TFM-C or TFM-C treatment. Scale bar 5µm. Panel b-c) 10µg of total protein were loaded for HERP Western blot analysis. Results were expressed as arbitrary units respect to the control at same time point. Error bars indicate the standard error. *P<0.05 by ANOVA test. D) Effect of TFM-C treatment (50µM) on the viability of BV2 cells. Apoptotic cells were measured by double propidium iodite (PI) and DAPI staining, and the percentage of damaged DNA and condensed chromatin was calculated at 24h of LPS/TFM-C treatment.

Previously, we have demonstrated that TFM-C inhibits secretion of the (hetero)dimeric IL-12 family cytokines IL-12, p40_2_ and IL-23 through a Ca^2+^-dependent mechanism involving chaperone-mediated cytokine retention in the ER coupled to degradation via HERP protein, and that TFM-C dramatically upregulates *HERP* gene expression in various cell lines [5,6,9]. In organotypic cultures, virtually no effect was seen on mRNA production of *HERP* at 6 and 12 hours of LPS/TFM-C treatment in compared with LPS stimulation (Figure 1B). To verify whether TFM-C is capable of inducing ER stress in microglia, the mouse microglial BV2 cell line was used. Specifically, we measured mRNA of HERP and IL-23p19, both of which are induced by ER stress [5,6,35]. BV2 cells were pre-treated with TFM-C (30 and 50 µM) for 2 hours and then stimulated with LPS for 3, 6, 12 and 24 hours in the presence or absence of TFM-C, which showed significant dose-dependent up-regulation of IL-23p19 and HERP (Figure 3B). Figure 3C shows that co-treatment of LPS with TFM-C led to increased accumulation of HERP in microglial cells (Figure 3C, panel a). In immunoblot, HERP protein levels were increased in BV2 cells at 12 hours of treatment in the presence of 50 µM of TFM-C plus LPS compared to LPS-only challenge (Figure 3C, panels b and c; Figure S5). To answer the question whether TFM-C is also able to block cytokine release from BV2 cells, IL-1β, IL-6 and TNF-α mRNA and secreted protein levels were analyzed (Figure 3A). TFM-C significantly decreased LPS-induced IL-1β mRNA and protein secretion starting at 6 hours of treatment. However, TFM-C exerted biphasic opposing effects on TNF-α mRNA levels at 3 (decrease) versus 24 (increase) hours compared to LPS-only challenge; of note, TFM-C suppressed secreted TNF-α protein levels throughout the duration of the experiment. For IL-6, no significant changes were observed in mRNA levels while the secreted protein was down-regulated at 12 hours of TFM-C treatment. To rule out occurrence of apoptotic cell death as contributing factor in suppression of cytokine secretion, cells were stained with DAPI and PI which is only incorporated by cells that have suffered membrane damage. TFM-C/LPS treatment decreased cell viability with approximately 4%, similar to LPS-only treated cells (Figure 3D). To identify additional UPR/ER stress-regulated genes potentially involved in altered cytokine folding/assembly/secretion, the effect of TFM-C on transcription of 252 genes belonging to UPR, autophagy and ubiquitination pathways was analyzed in HEK-293 cells (Figure S2A). We identified 36 genes with a fold increase > 2.5 and confirmed *HERP* as the gene showing the fifth highest degree of upregulation by TFM-C (fold increase of 9.1 in the UPR PCR Arrays). *DDIT3* (CHOP), a primordial ER stress response gene [13,36], was identified with some distance as the gene showing the highest degree of induction by TFM-C (36-fold). Further analysis of the kinetics of upregulation by TFM-C of 5 of these genes, *PARK2, ARMET, FBXO4, RAB24* and *MAP1LC3B*, in addition to *HERP*, was performed in both HEK-293 and BV2 cells (Figure S2B and S2C). At the protein level, only HERP, MAP1LC3B and ARMET showed clear changes in the kinetics of their production in HEK-293 cells. In BV2 cells, TFM-C significantly decreased PARK2 expression at 3 and 12 hours, and increased RAB24 expression at 3 hours both compared with LPS-treated cells. No modification in MAP1LC3B and ARMET protein expression was observed (Figure S2D). Thus, TFM-C induces a gene expression signature predominantly enriched for UPR/ER stress response and ubiquitination pathway genes, including to a lesser extent also some genes involved in autophagy (Figure S2A). Pending mechanistic studies, this suggests that its suppressive effect on cytokine secretion may be functionally related to alterations in protein transit and turnover pathways, as suggested in our previous work [5,6,9,17]. 

### TFM-C induces intracellular retention of TNF-α in BV2 microglia but increases its secretion from primary astrocytes

**Figure 4:**
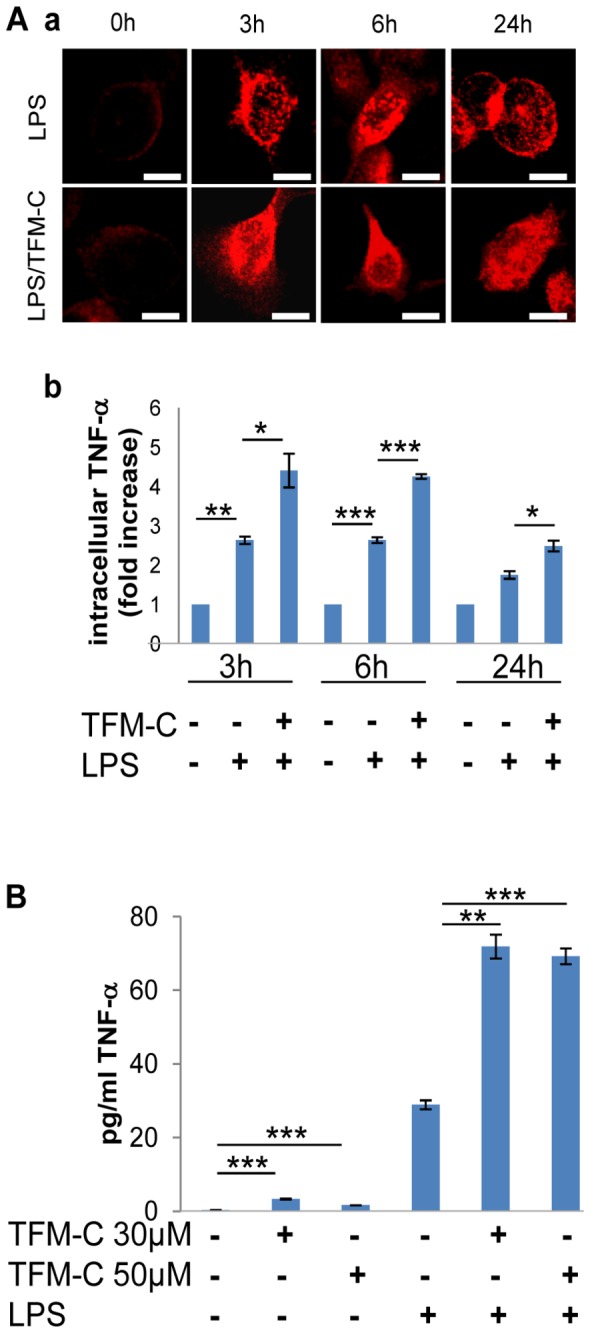
Intracellular localization of TNF-α in BV2 cell line and TNF-α secretion in primary astrocytes. A) BV2 cells were treated with TFM-C (50µM) for 2h and stimulated with LPS (1µg/ml) for 3, 6 and 24h in presence or absence of TFM-C. Panel a) Staining for TNF-α in permeabilized cells. Scale bar 5µm. Panel b) Intracellular localization of TNF-α by flow cytometry. Results are expressed as fold increase compared to the control at the same time point. B) TNF-α release in astrocyte cultures. Astrocytes were treated with TMF-C (30 or 50µM) for 24h or pre-treated with TMF-C for 2h and then stimulated with LPS (1µg/ml) in presence or absence of TFM-C for 24h and then analyzed by ELISA. Error bars indicate the standard deviation. *P<0.05, **P<0.01, ***P<0.001 by ANOVA test.

As demonstrated in Figure 3A, TFM-C reduces secreted levels of IL-1β and TNF-α from BV2 microglial cells. To clarify whether TNF-α was retained intracellularly, an immunofluorescence approach was used to monitor subcellular distribution of TNF-α in the ER/Golgi compartment (in permeabilized cells) or at the plasma membrane (in nonpermeabilized cells). BV2 cells treated with LPS exhibited pronounced intracellular TNF-α staining at 3, 6 and 24 h (Figure 4A, panel a). Co-treatment with TFM-C further enhanced intracellular staining of TNF-α, which was validated by flow cytometry and densitometric analysis (Figure 4A panels a and b, and Figure S3). No significant differences were observed in TNF-α staining at the plasma membrane in nonpermeabilized cells between TFM-C/LPS- and LPS-treated cells (Figure S4). This data suggests that the enhanced levels of secreted TNF-α in TFM-C/LPS-treated organotypic cerebellar cultures (Figure 1A) is unlikely to originate from microglial cell sources. Therefore, we analyzed TNF-α production from isolated primary cortical astrocytes, another LPS-inducible natural source of TNF-α in the Central Nervous System (CNS) [37]. As shown in Figure 4B, astrocyte cultures treated with TFM-C alone displayed marginally increased TNF-α release compared with untreated cultures. However, treatment with LPS/TFM-C induced a 2-fold increase in TNF-α secretion compared with the cultures stimulated with LPS only. This result demonstrates that resident astrocytes in the cerebellar organotypic cultures rather than microglia may constitute the source of TNF-α by TFM-C (Figure 1A). 

### TFM-C suppresses EAE

**Figure 5:**
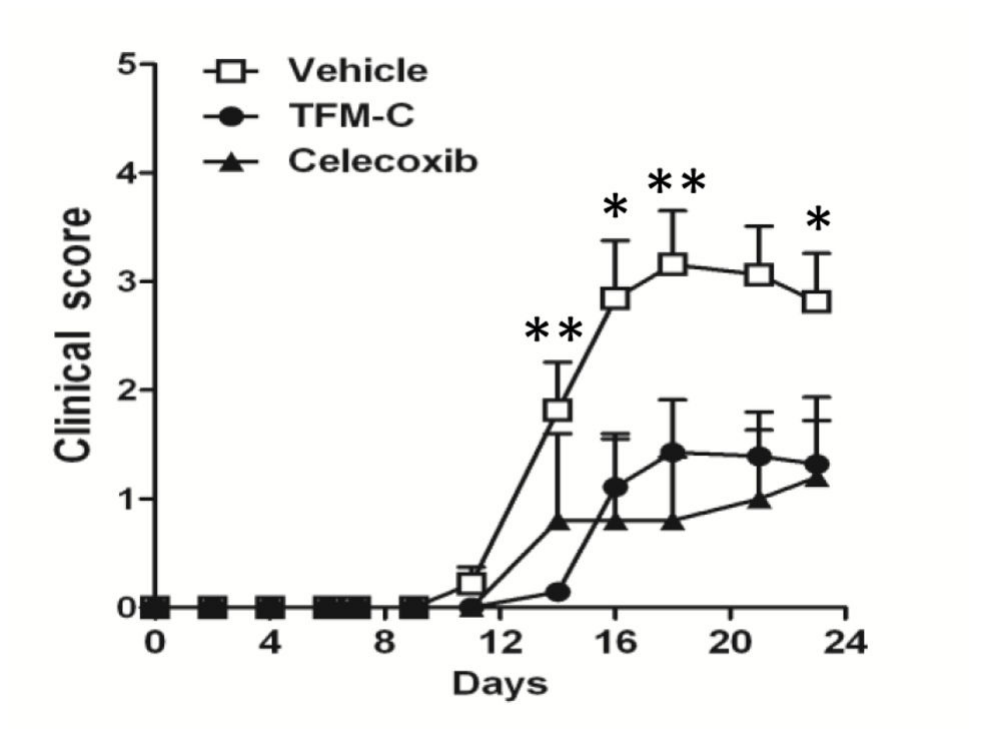
The effect of TFM-C on EAE. Clinical EAE scores of female B6 mice following immunization with MOG35-55. The mice were injected i.p. with 10 µg/kg TFM-C (closed circles), celecoxib (closed triangles) or vehicle (open squares) every other day starting on the day of immunization. *P<0.05 TFM-C-treated versus vehicle-treated group. Results shown are the mean + SEM of 14-16 mice per group in TFM-C- or vehicle-treated groups and of 5 mice in the celecoxib-treated group. The data shown are pooled data from three similar experiments.

Previously we demonstrated that celecoxib inhibited EAE in COX-2-deficient mice [16], associated with reduced MOG-specific Th1 cytokines. Thus, TFM-C treatment might be inferred to exert an effect on autoreactive T cells by suppressing production of these cytokines. We examined the effect of TFM-C on EAE induced by immunization with MOG derived peptide, MOG35-55, in which disease development largely depends on encephalitogenic T cells. The administration of TFM-C reduced the severity of EAE when compared with the control group (Figure 5). The incidence of disease in the control group was 93.8% and TFM-C reduced the incidence to 57.1%. The average day of onset was 15.2±3.1 in control group and 16.4 ±2.5 in the TFM-C-treated group. This result indicates that TFM-C limits the severity of disease showing similar effects to celecoxib [16].

### TFM-C suppresses IFN-γ and IL-17 production from MOG-reactive T cells

**Figure 6:**
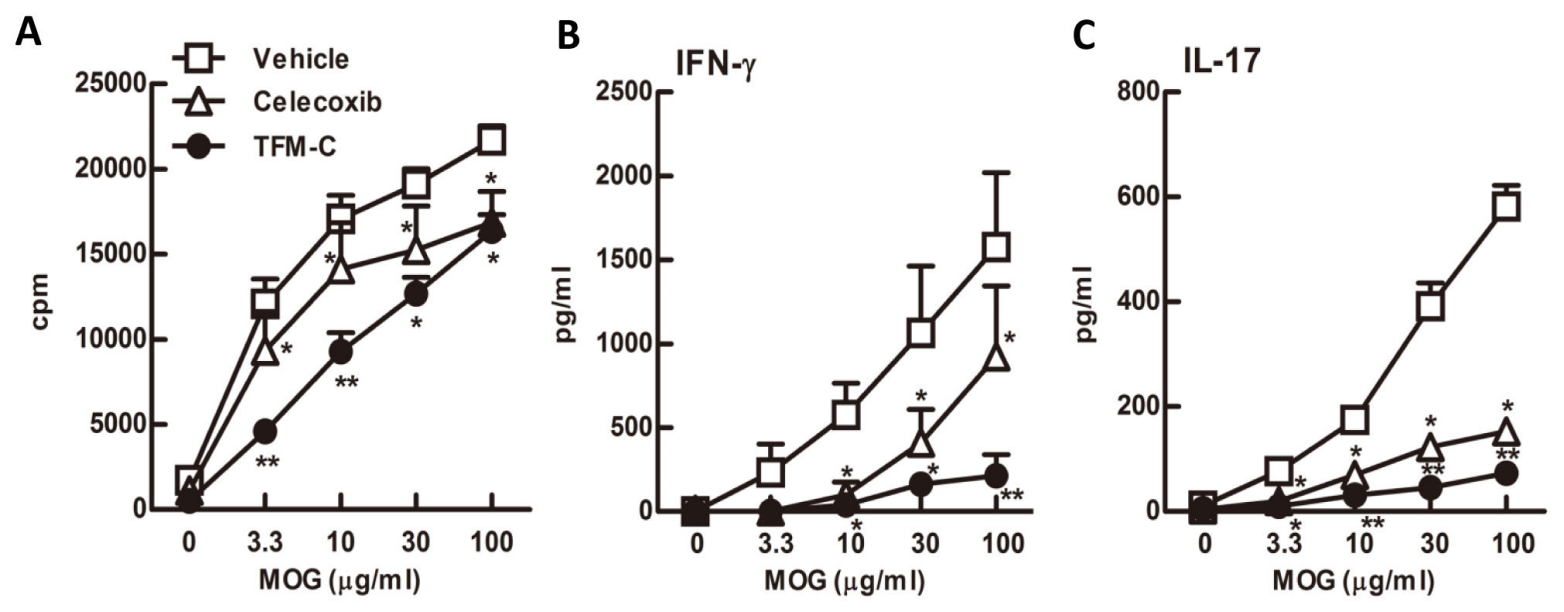
MOG35-55 specific T-cell responses in mice treated with TFM-C. A) Popliteal and inguinal lymph node cells from TFM-C-, celecoxib-treated or control mice were incubated in the presence of MOG35-55 for 48h. Proliferative responses were determined by the uptake of [^3^H] thymidine. B and C). The levels of IFN-γ and IL-17 in culture supernatants were measured by ELISA. The data shown are from a single experiment representative of three similar experiments. Error bars represent + SEM of 3 mice per group. *P<0.05 compared with control group, **P<0.05 compared with both control and celecoxib-treated groups.

We next examined the effect of TFM-C on MOG35-55-specific T cell responses by *ex vivo* re-challenge with MOG35-55 peptide 10 days after immunization. The proliferative response to MOG35-55 was slightly reduced in celecoxib-treated mice (Figure 6A). TFM-C exhibited a stronger inhibitory effect on MOG35-55-reactive T cell proliferation, even though the overall effect of TFM-C on proliferation was also limited (Figure 6A). Compared to control cells, lymph node cells from MOG35-55-primed TFM-C-treated mice produced significantly lower levels of IFN-γ; this was also true for lymph node cells from celecoxib-treated mice albeit to lesser degree (Figure 6B). Furthermore, the level of IL-17 was also significantly reduced in both TFM-C and celecoxib-treated mice compared to that in control mice (Figure 6C). The level of IL-4 was below detection limits (

< 5 pg/ml) (data not shown). These results indicate that TFM-C suppresses MOG35-55-specific T cell proliferation and the production of IL-17 and IFN-γ, and that the suppressive effects of TFM-C on MOG35-55-specific T cell responses are stronger than those of celecoxib.

### TFM-C inhibits the production of IL-23 and inflammatory cytokines by dendritic cells

**Figure 7:**
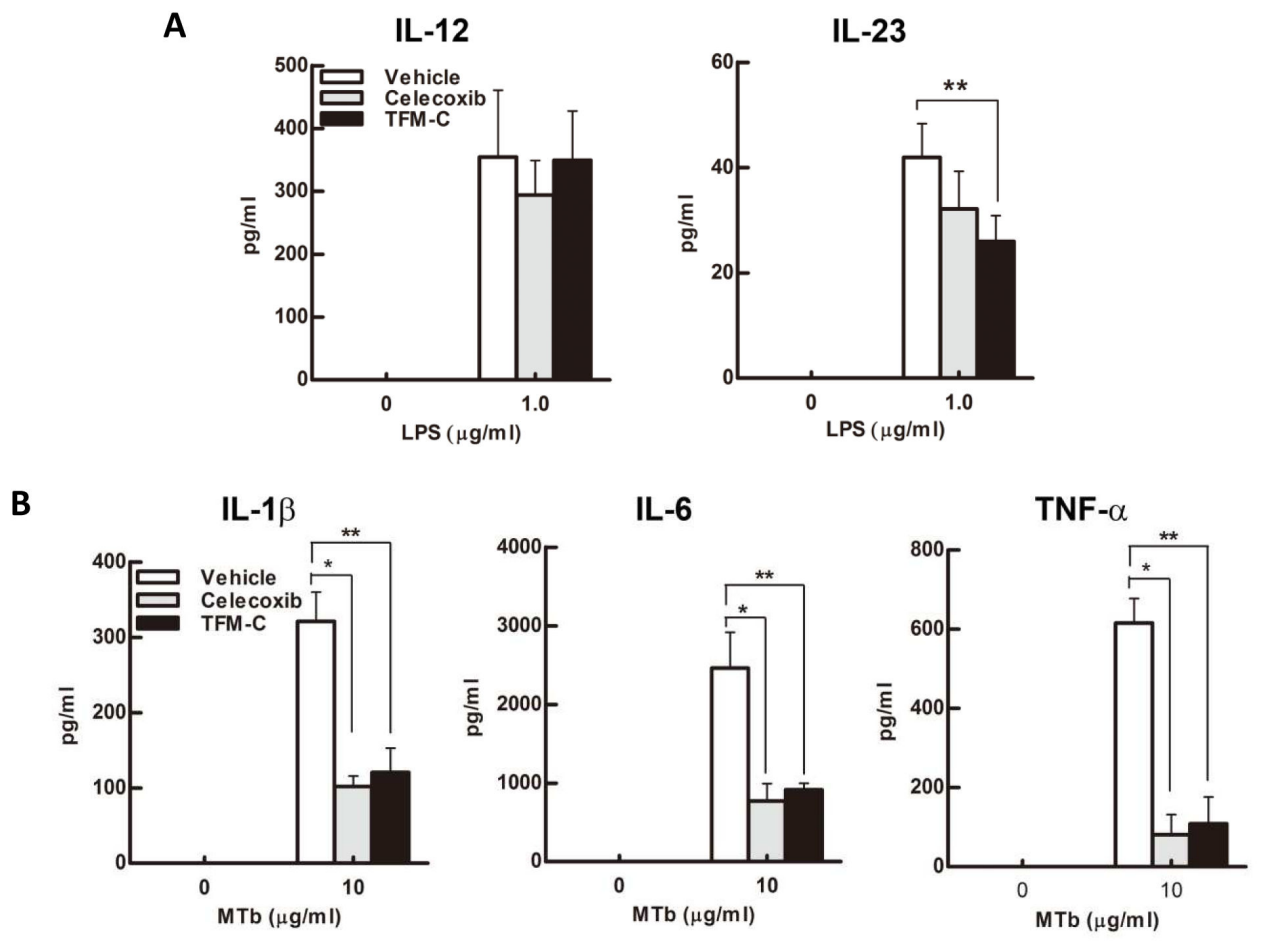
The effect of TFM-C on cytokine production from BMDCs. BMDCs were incubated in TFM-C, celecoxib or vehicle for 16h and subsequently stimulated with LPS (0.1µg/ml) (A) or heat killed H37Ra Mtb (10µg/ml) (B) in the presence of TFM-C, celecoxib or vehicle. Cytokines were detected by ELISA. IL-12, IL-1β or IL-6 were measured 24h after stimulation. IL-23 and TNF-α were measured 6h after stimulation. The data shown are pooled from two similar experiments. Error bars represent + SEM. *P<0.05 control versus TFM-C celecoxib-treated group, **P<0.05 control versus TFM-C -treated group.

To assess the effect of TFM-C on natural production of IL-12/IL-23, we stimulated Bone Marrow-derived Dendritic Cells (BMDCs) with LPS with or without TFM-C or celecoxib, and measured IL-12 and IL-23 in the culture supernatants. As shown in Figure 7A, IL-23 production was significantly suppressed in the presence of TFM-C. IL-23 suppression was also observed for BMDCs in the presence of celecoxib although the difference did not reach statistical significance. In contrast, neither compound inhibited IL-12 production. To examine the effect on the production of inflammatory cytokines, we stimulated BMDCs with heat-killed *Mtb*, a potent IL-1 stimulator, with or without TFM-C or celecoxib. As shown in Figure 7B, IL-1β, IL-6 and TNF-α production were inhibited in the presence of TFM-C or celecoxib. These results indicate that TFM-C inhibits the production of IL-23 and inflammatory cytokines by BMDCs.

## Discussion

In this study, we have tested a non-coxib analogue of celecoxib, TFM-C, both in a model of neuroinflammation consisting of organotypic cultures challenged with LPS [32] and in EAE. The former model recapitulates several events that occur during brain inflammation, including microglia activation followed by cytokine release and oxidative stress, demyelination and axonal damage and permits to study the effect and the mechanism of new treatments for neuroinflammatory disorders [31,32]. Of note, TFM-C significantly decreased axonal damage and oxidative stress in organotypic cultures associated with suppression of IL-1β, IL-12 and IL-17 secretion but enhanced that of TNF-α and RANTES. Similarly to celecoxib in EAE, TFM-C reduced infiltration in CNS, demyelination and production of IFN-γ and IL-17. Further analysis showed that astrocytes cultures treated with TFM-C increased TNF-α release. Moreover, we observed a 2-fold increase in the TNF-α secretion when the astrocytes were co-treated with LPS/TFM-C for 24 hours compared with the cultures stimulated with LPS only. In contrast, in the BV2 microglia cell line, TFM-C suppressed secreted TNF-α protein levels. An explanation for these opposite effects should be sought in the differential response of astrocytes and microglia to modulation of intracellular calcium homoeostasis through selectively inhibiting ER Ca^2+^-ATPases by celecoxib or TFM-C [5,38]. Astrocytes possess an intracellular Ca^2+^-dependent excitability leading to glutamate release [39-44]. For example, A23187, a Ca^2+^-dependent ER stressor, induces elevation of calcium levels, thus increasing glutamate release [45]. In addition, stimulation of astrocytes by glutamate or ATP induces a glutamatergic response accompanied by TNF-α release [46]. However, ionomycin, another Ca^2+^-dependent ER stressor, does not induce cytokine release in microglia, indicating that in microglia Ca^2+^ perturbation is not sufficient to induce cytokines secretion [47]. These data suggest that the resident astrocytes and microglia in our cerebellar organotypic cultures respond differentially to Ca^2+^ perturbation by TFM-C in terms of TNF-α secretion. 

We also observed a significant increase of RANTES levels in organotypic cultures treated with TFM-C. RANTES is a chemokine that promotes the recruitment and activation of inflammatory cells such as monocytes, lymphocytes, mast cells and eosinophils [48-51]. RANTES also attracts memory T cells, promoting the formation of mononuclear infiltrates characteristic of MS [49,52]. Moreover, it has been described that astrocytes can generate RANTES after stimulation with TNF-α, IL-1β and IFN-γ. Actually, RANTES can be induced only by TNF-α alone in rat primary astrocytes [53]. 

The role of TNF-α in the pathogenesis of inflammatory demyelinating disease of the central nervous system has been demonstrated in rodents [54] and in humans [55-57]. Transgenic mice that constitutively express TNF-α in the CNS can trigger the development of a chronic inflammatory demyelinating disease [58]. TNF-α is synthesized as a transmembrane precursor protein (tmTNF) and its cytoplasmic tail is cleaved by the TNF-α-converting enzyme (TACE) to release a soluble TNF-α (sTNF). These two forms of TNF-α interact with the receptors, tumor necrosis factor 1 (TNFR1) and 2 (TNFR2). TNFR1 is expressed in all cell types and is preferentially bound by sTNF whereas TNFR2 is expressed by endothelial cells and immune cells and especially bound by tmTNF [59]. The activation of TNFR1 can induce either activation of NFκB or apoptosis via caspase 8 and 3 [60]. The binding to TNFR2 induces proinflammatory and survival signaling pathways [61]. Comparison of TNFR1 and TNFR1/TNFR2 knockout and wild-type mice, shows that EAE symptoms were milder or absent in the knockout animals. In contrast, in TNFR2-deficient mice the symptoms were enhanced and associated with high inflammation and demyelination [62,63]. Remyelinated function was conferred to TNFR2 whereas demyelination to TNFR1 [64]. Recently, we have shown that demyelination was significantly attenuated in cerebellar cultures challenged with LPS pretreated with Fc-TNFR1 recombinant protein [32]. In particular, we found that myelin damage and oligodendrocyte loss were promoted by pro-inflammatory cytokines such as TNF-α. Probably, the observation that the TFM-C treatment had no effect on demyelination in organotypic culture model could be explained by increased astrocytic TNF-α, that in turn induces TNFR1 activation. Moreover, immune cells expressing TNFR2 are lacking in organotypic cultures. In contrast, in the same model TFM-C exerts beneficial effects restoring oxidative stress and axonal damage induced by LPS. 

In the EAE model, although the treatment with TFM-C failed to completely inhibit the development of EAE, TFM-C significantly reduced the severity of the disease decreasing the incidence from 93.8% to 57.1%. Moreover, TFM-C suppressed the production of IL-23 and inflammatory cytokines, IL-1β, IL-6 and TNF-α from dendritic cells and this suppressive effect was strong enough to decrease the subsequent production of IL-17 and IFN-γ from lymph node cells upon stimulation with MOG. The differential effects observed on IL-23 inhibition by celecoxib vs TFM-C suggest a beneficial effect of the latter via inhibition of the Th17 responses. In addition to the suppression of IL-23, blockage of IL-6 and IL-1β, two cytokines that affect development of Th17 cells, would also contribute to the suppression of IL-17 [65,66]. TNF-α, IL-1β and IL-6 can increase the permeability of the blood-brain barrier (BBB) [67]. IL-6 induces leukocyte chemoattraction to the endothelium, as well as lymphocyte activation. IL-6 is found in high concentrations in active MS lesions [68,69]. In EAE, IL-6 induction is found in the spinal cord [70]. The detrimental role of IL-6 in this model, associated with BBB permeability, is evidenced in IL-6-deficient mice that are resistant to the pathophysiological alterations [71]; on the other hand, mice overexpressing astrocytic IL-6 showed indeed breakdown of the BBB [72]. Moreover, the inhibition of IL-6 in BMDCs and macrophages [17] treated with TFM-C might suggest that the beneficial effect of TFM-C in EAE can depend on its ability to reduce BBB permeability.

The different effect of TFM-C observed in CNS-derived cells versus lymph node-derived cells may reside in incomplete penetration of TFM-C into CNS. In the *in vivo* system, TFM-C may exert a beneficial effect on peripheral cells, reducing proliferation and pro-inflammatory cytokine secretion, thus ameliorating disease severity. In contrast, when CNS cells are exposed directly to TFM-C, such as in the organotypic, astrocyte or microglia cultures, these cells may respond to Ca^2+^ perturbation differentially by either increasing or decreasing pro-inflammatory cytokine secretion. However, it remains to be determined whether TFM-C penetrates into the intact CNS following i.p. injection. If not, the main cellular mediators of its cytokine-modulating effects are likely to be the peripheral immune cells. This could explain why TFM-C failed to inhibit completely the development of EAE. In summary, we have described a new drug with potential beneficial effects for MS. The therapeutic niche covered by TFM-C may include beneficial effects in both innate and adaptive immunity, which is incompletely covered by current therapies [73]. In this sense, the therapy can be defined as acting in the interface between immunomodulation and neuroprotection. Thus, design of further non-coxib celecoxib analogues with improved activity profiles may be warranted.

## Supporting Information

Figure S1 **Effect of TFM-C on MHCII protein expression.** Organotypic cultures were stimulated with LPS for 24h or pre-treated with TFM-C (50µM) for 2h and then stimulated with LPS/TFM-C for 24h. Immunofuorescence for Iba1 (red) and MHCII (green) was performed. White boxes show higher magnifications of merged images. Scale bar 50µm.
(TIF)

Figure S2 **Effect of TFM-C on expression of genes belonging to Unfolded Protein Response (**U**), Ubiquitination (Ub) and Autophagy (**A**) pathways in** **HEK-293 cells and BV2 cells**. A) HEK-293 cells were treated with TFM-C (50µM) for 12h and U, Ub and A PCR arrays were performed. The genes represented in the table are those with a fold increase higher than 2.5 compared with control samples and are ranked according to expression level (high to low). B) HEK-293 cells were treated with TFM-C (50µM) for different times and RT-QPCR was performed. Selected genes (*PARK2, FBXO4, RAB24, MAP1LCB3, HERP* and *ARMET*) were validated in three independent experiments. The levels of mRNA are shown as 2^-∆∆Ct^ compared with the level of baseline condition (-) and normalized with the housekeeping geneGlyceraldehyde 3-phosphate dehydrogenase. Asterisks indicate significant differences at *P<0.05 by Student’s test. C) 20µg of total protein were loaded for PARK2, FBXO4, RAB24, MAP1LCB3, HERP, ARMET and tubulin Western blot analysis D) BV2 cells were pre-treated with TFM-C (30 or 50µM) for 2h, then stimulated with LPS for different times and 10µg of total protein were loaded for Western blot analysis. Results were expressed as arbitrary units compared to the control at same time point. All values represent the averages of three independent experiments. Error bars indicate the standard error. *P<0.05, **P<0.01, ***P<0.001 by ANOVA test.
(TIF)

Figure S3 **Quantification of intracellular TNF-α in BV2 cell line.** The cells were treated as described in Figure 5 and the fluorescence intensity was analyzed via densitometry using a fluorescence microscope by calculating mean grey value (pixel intensity) normalized by fixed area (ROI=Region Of Interest; Image J Software). For each condition ten cells/field were analyzed for a total of 4 fields and 40 cells. Error bars indicate the standard error.
(TIF)

Figure S4 **Quantification of TNF-α in plasma membrane of BV2 cell line.** BV2 cells were treated with TFM-C (50µM) for 2h and stimulated with LPS (1µg/ml) for 3, 6 and 24h in presence or absence of TFM-C. The cells were fixed with PFA and stained for TNF-α. A) Fluorescence intensity was analyzed by densitometry calculating mean pixel intensity normalized by ROI fixed area (Image J Software). For each condition ten cells/field were analyzed for a total of 4 fields and 40 cells. Error bars indicate the standard error. B) Staining for TNF-α in non-permeabilized cells. Scale bar 5µm.
(TIF)

Figure S5 **Normalization of protein expression.** BV2 cells were treated with TFM-C (50µM) for different times and 10µg of total protein were loaded in Stain-free Precast Gels and then transferred to nitrocellulose membrane. A) Total protein was visualized by UV excitation. B) Tubulin protein expression analyzed by Western blot. C) Quantification of HERP band intensity normalized with total protein loaded. D) Quantification of HERP band intensity normalized with Tubulin protein. The values represent the averages of three independent experiments. Significant differences at *P<0.05 and **P<0.01 compared with control (0h) by Student’s test.
(TIF)
