# Supplementary figures and images for: A Trifluoromethyl Analogue of Celecoxib Exerts Beneficial Effects in Neuroinflammation
Source: PLoS One. 2013 Dec 11;8(12):e83119. doi: 10.1371/journal.pone.0083119 (PMC3859644; doi:10.1371/journal.pone.0083119)

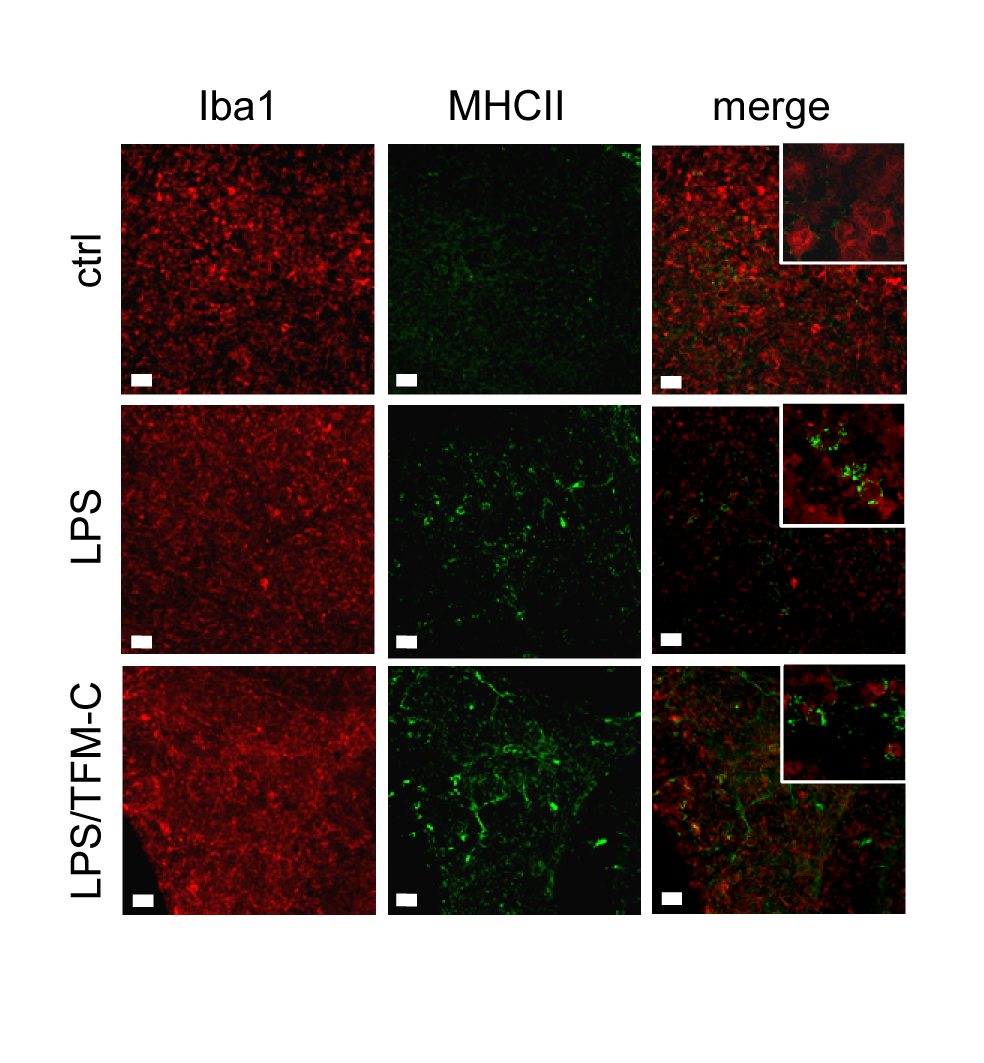

Supplement: Figure S1 — Effect of TFM-C on MHCII protein expression. Organotypic cultures were stimulated with LPS for 24h or pre-treated with TFM-C (50µM) for 2h and then stimulated with LPS/TFM-C for 24h. Immunofuorescence for Iba1 (red) and MHCII (green) was performed. White boxes show higher magnifications of merged images. Scale bar 50µm. (TIF) [file pone.0083119.s001.tif]

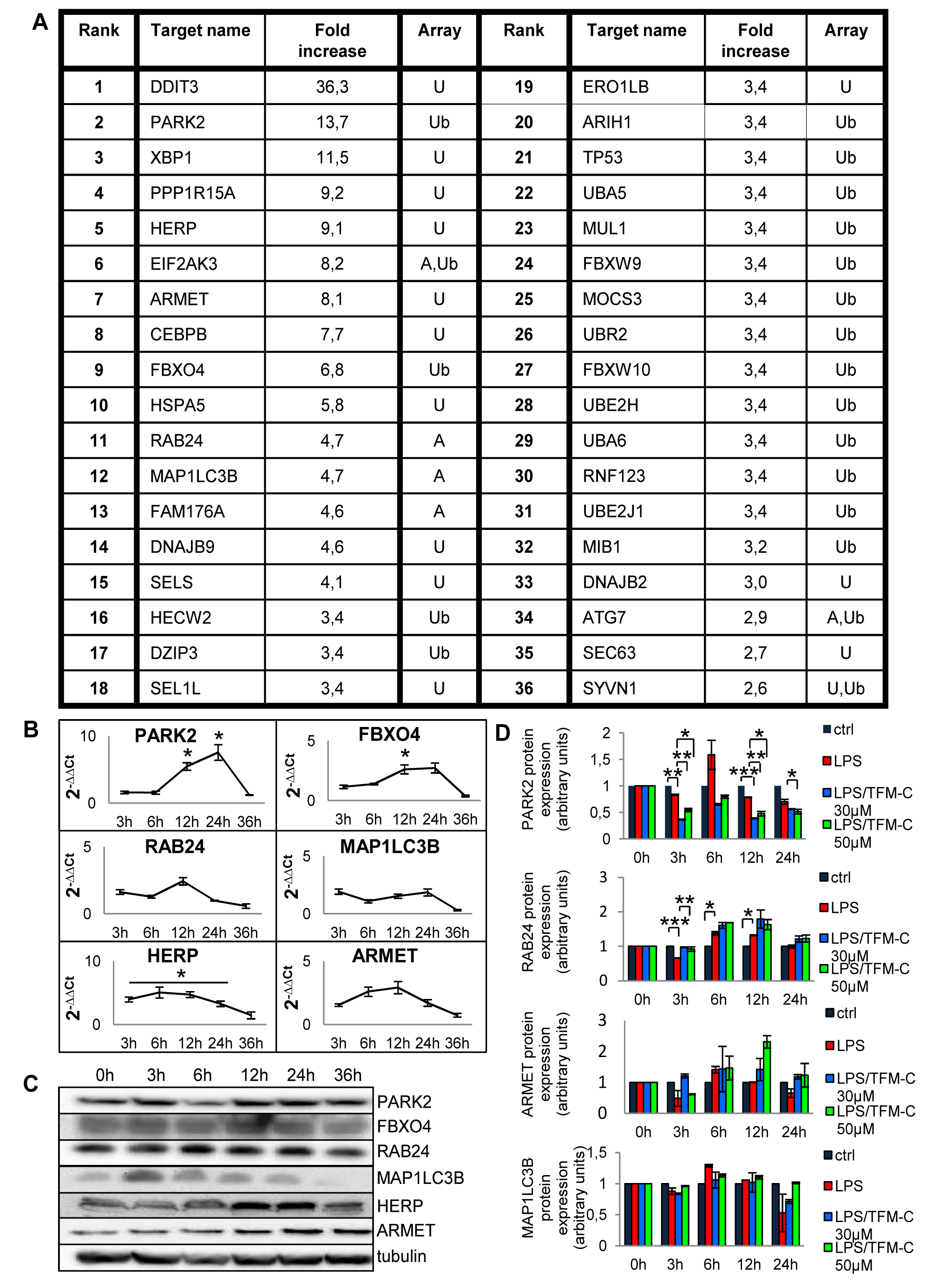

Supplement: Figure S2 — Effect of TFM-C on expression of genes belonging to Unfolded Protein Response (U), Ubiquitination (Ub) and Autophagy (A) pathways in HEK-293 cells and BV2 cells. A) HEK-293 cells were treated with TFM-C (50µM) for 12h and U, Ub and A PCR arrays were performed. The genes represented in the table are those with a fold increase higher than 2.5 compared with control samples and are ranked according to expression level (high to low). B) HEK-293 cells were treated with TFM-C (50µM) for different times and RT-QPCR was performed. Selected genes (PARK2, FBXO4, RAB24, MAP1LCB3, HERP and ARMET) were validated in three independent experiments. The levels of mRNA are shown as 2-∆∆Ct compared with the level of baseline condition (-) and normalized with the housekeeping geneGlyceraldehyde 3-phosphate dehydrogenase. Asterisks indicate significant differences at *P<0.05 by Student’s test. C) 20µg of total protein were loaded for PARK2, FBXO4, RAB24, MAP1LCB3, HERP, ARMET and tubulin Western blot analysis D) BV2 cells were pre-treated with TFM-C (30 or 50µM) for 2h, then stimulated with LPS for different times and 10µg of total protein were loaded for Western blot analysis. Results were expressed as arbitrary units compared to the control at same time point. All values represent the averages of three independent experiments. Error bars indicate the standard error. *P<0.05, **P<0.01, ***P<0.001 by ANOVA test. (TIF) [file pone.0083119.s002.tif]

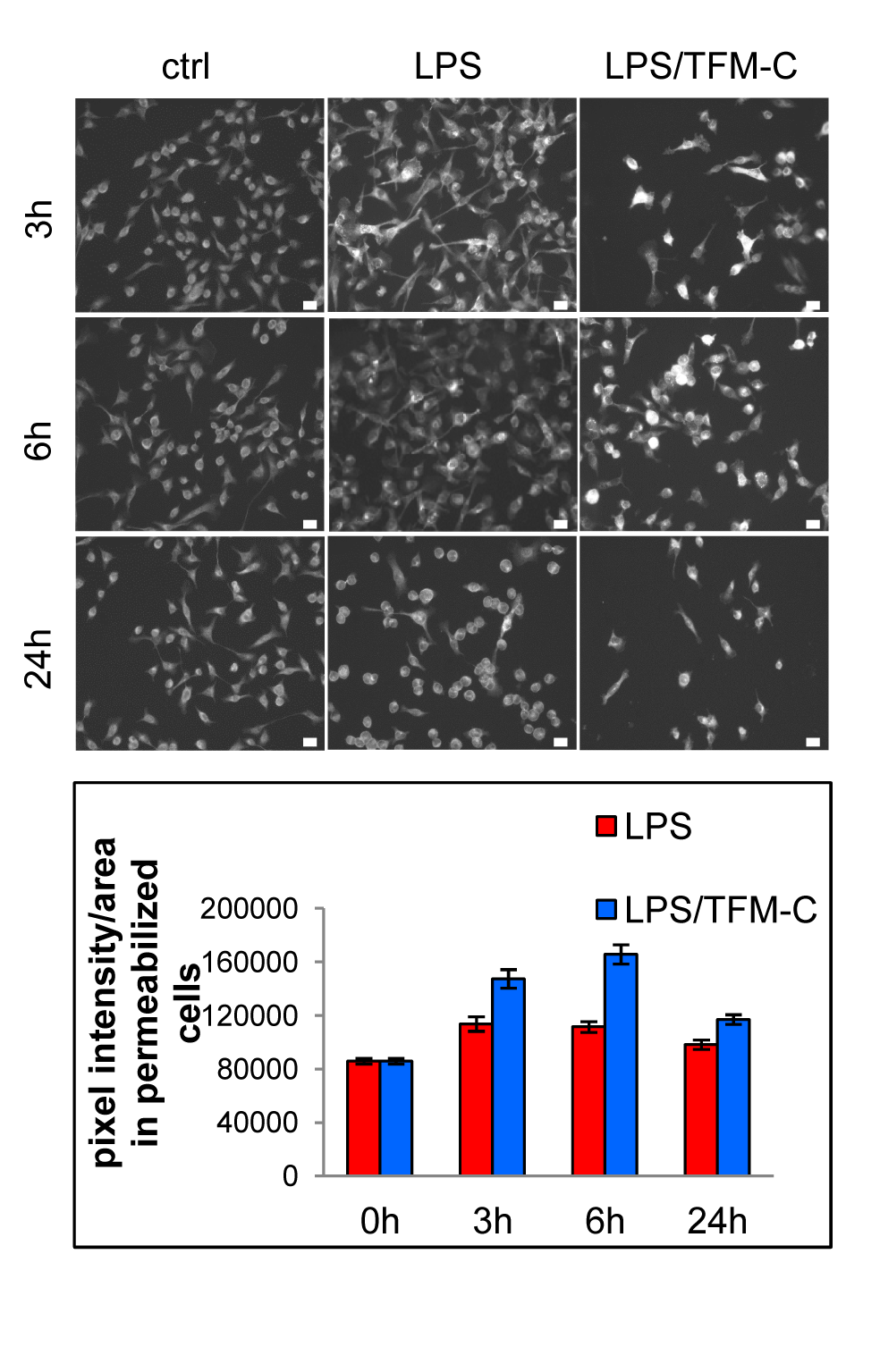

Supplement: Figure S3 — Quantification of intracellular TNF-α in BV2 cell line. The cells were treated as described in Figure 5 and the fluorescence intensity was analyzed via densitometry using a fluorescence microscope by calculating mean grey value (pixel intensity) normalized by fixed area (ROI=Region Of Interest; Image J Software). For each condition ten cells/field were analyzed for a total of 4 fields and 40 cells. Error bars indicate the standard error. (TIF) [file pone.0083119.s003.tif]

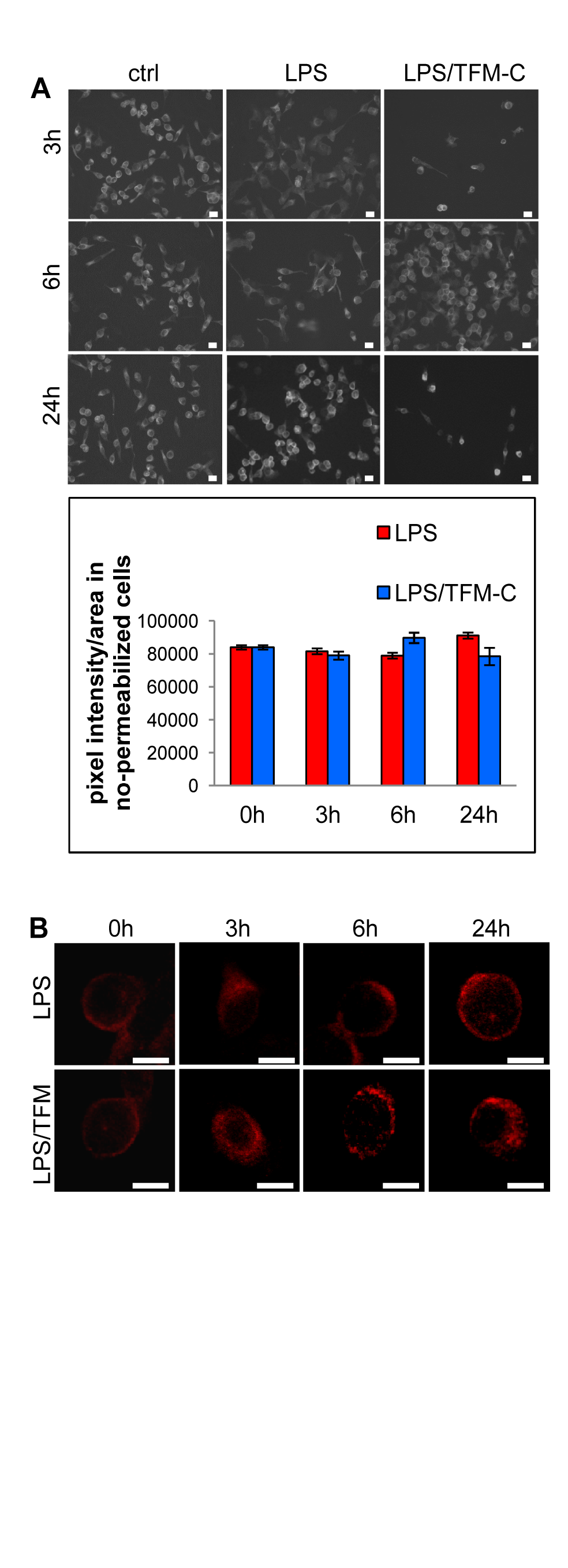

Supplement: Figure S4 — Quantification of TNF-α in plasma membrane of BV2 cell line. BV2 cells were treated with TFM-C (50µM) for 2h and stimulated with LPS (1µg/ml) for 3, 6 and 24h in presence or absence of TFM-C. The cells were fixed with PFA and stained for TNF-α. A) Fluorescence intensity was analyzed by densitometry calculating mean pixel intensity normalized by ROI fixed area (Image J Software). For each condition ten cells/field were analyzed for a total of 4 fields and 40 cells. Error bars indicate the standard error. B) Staining for TNF-α in non-permeabilized cells. Scale bar 5µm. (TIF) [file pone.0083119.s004.tif]

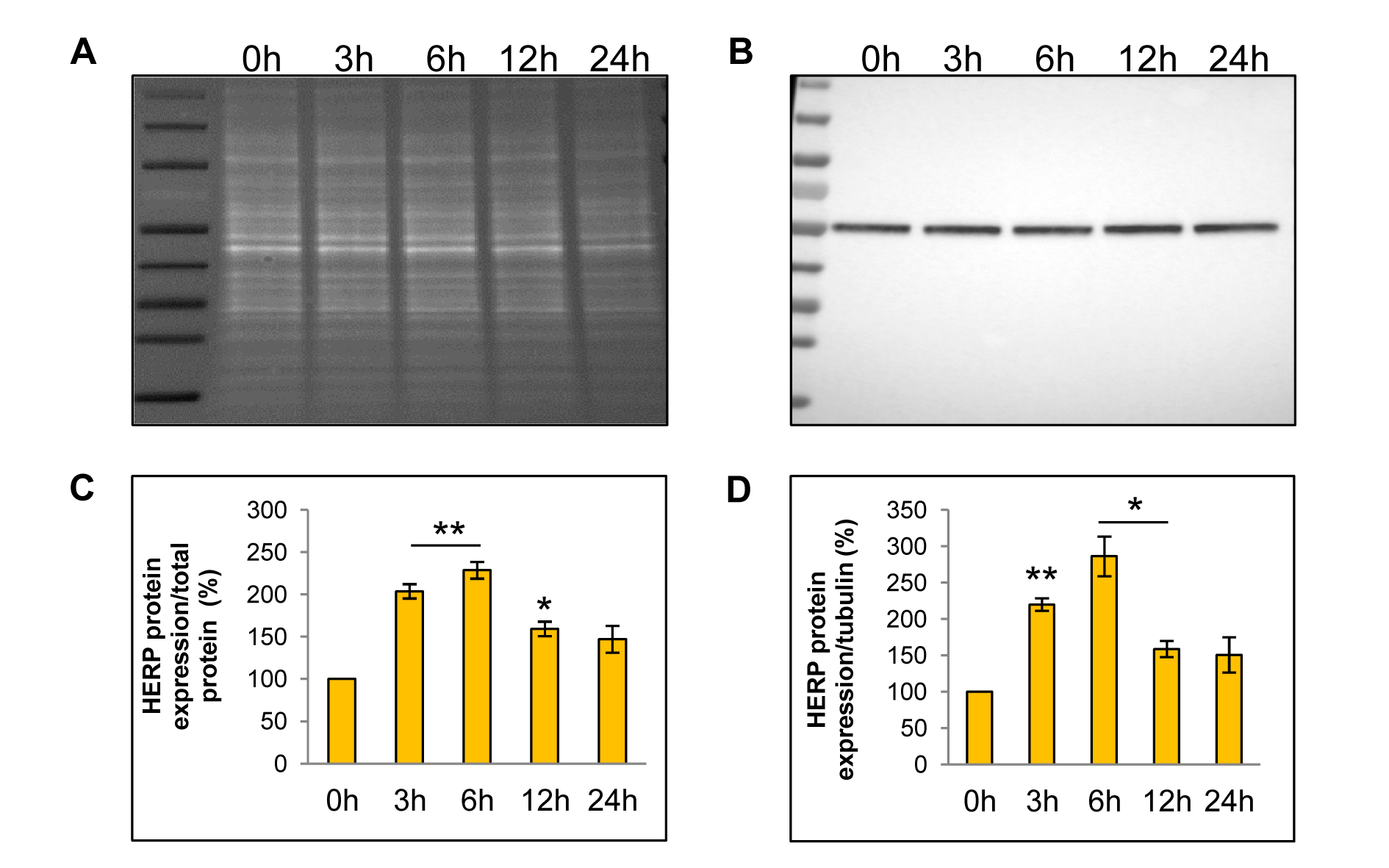

Supplement: Figure S5 — Normalization of protein expression. BV2 cells were treated with TFM-C (50µM) for different times and 10µg of total protein were loaded in Stain-free Precast Gels and then transferred to nitrocellulose membrane. A) Total protein was visualized by UV excitation. B) Tubulin protein expression analyzed by Western blot. C) Quantification of HERP band intensity normalized with total protein loaded. D) Quantification of HERP band intensity normalized with Tubulin protein. The values represent the averages of three independent experiments. Significant differences at *P<0.05 and **P<0.01 compared with control (0h) by Student’s test. (TIF) [file pone.0083119.s005.tif]
